# Synergistic Effect and Characterization of Graphene/Carbon Nanotubes/Polyvinyl Alcohol/Sodium Alginate Nanofibrous Membranes Formed Using Continuous Needleless Dynamic Linear Electrospinning

**DOI:** 10.3390/nano9050714

**Published:** 2019-05-08

**Authors:** Ting-Ting Li, Yanqin Zhong, Mengxue Yan, Wei Zhou, Wenting Xu, Shih-Yu Huang, Fei Sun, Ching-Wen Lou, Jia-Horng Lin

**Affiliations:** 1 Innovation Platform of Intelligent and Energy-Saving Textiles, School of Textile Science and Engineering, Tianjin Polytechnic University, Tianjin 300387, China; tingtingli@tjpu.edu.cn (T.-T.L.); 1831015063@stu.tjpu.edu.cn (Y.Z.); 13032210827@163.com (M.Y.); zw13819064983@163.com (W.Z.); 2180312@mail.dhu.edu.cn (W.X.); 1720011046@stu.tjpu.edu.cn (F.S.); 2 Tianjin and Education Ministry Key Laboratory of Advanced Textile Composite Materials, Tianjin Polytechnic University, Tianjin 300387, China; 3 Fujian Key Laboratory of Novel Functional Fibers and Materials, Minjiang University, Fuzhou 350108, China; cwlou@asia.edu.tw; 4 Department of Chemical Engineering and Materials, Ocean College, Minjiang University, Fuzhou 350108, China; 5 Department of Bioinformatics and Medical Engineering, Asia University, Taichung 41354, Taiwan; 6 Department of Medical Research, China Medical University Hospital, China Medical University, Taichung 40402, Taiwan; 7 College of Textile and Clothing, Qingdao University, Shandong 266071, China; 8 Laboratory of Fiber Application and Manufacturing, Department of Fiber and Composite Materials, Feng Chia University, Taichung 40724, Taiwan; 9 School of Chinese Medicine, China Medical University, Taichung 40402, Taiwan; 10 Department of Fashion Design, Asia University, Taichung 41354, Taiwan

**Keywords:** needleless electrospinning, synergistic effect, graphene (Gr), carbon nanotubes (CNT), sodium alginate (SA), PVA

## Abstract

In this study, a self-made continuous needleless dynamic linear electrospinning technique is employed to fabricate large-scale graphene (Gr)/carbon nanotubes (CNT)/polyvinyl alcohol (PVA)/sodium alginate (SA) nanofibrous membranes. The synergistic effect of Gr and CNT fillers in the PVA/SA membrane is explored in depth by changing the volume ratio (*v*/*v*) of Gr and CNT as 10:0, 8:2, 6:4, 4:6, 2:8, and 0:10. Microstructure, functional group, conductivity, and hydrophilicity of PVA/SA/Gr/CNT membranes was characterized. Results show that the linear electrode needleless electrospinning technique can be spun into 200-nm diameter fibers. The PVA/SA/Gr/CNT fibrous membrane has good hydrophilicity and thermal stability. A Gr/CN ratio of 6:4 possessed the optimal synergistic effect, which showed the lowest surface resistivity of 2.53 × 10^3^ Ω/m^2^. This study will provide a reference for the large-scale preparation of nanofibrous membrane used as a artificial nerve conduit in the future.

## 1. Introduction

With the rise of the number of patients with impaired nerves every year, nerve conduits are used to bridge the broken end of nerves with an attempt to conduct and accelerate the regeneration of nerves. Thus, the nervous conduits have received a great deal of attention [1]. There are a great variety of studies on artificial nerve conduits currently. They are required to satisfy the safety, biocompatibility, biodegradability, biopermeability, appropriate mechanical strength, etc.

As a commonly used biodegradable polymer, polyvinyl alcohol (PVA) processed with hydrolysis polyvinyl acetate features high hydrophilicity, non-toxicity, and biocompatibility [2,3,4]. Sodium alginate (SA) is a kind of linear polysaccharides from brown algae, and also have higher biocompatibility and biodegradability [5]. These two polymers are usually applied to tissue engineering and drug delivery [6,7]. Moreover, when nerve cells proceed message passing, they are dependent on action potential generated by synapses. This process is a conversion of chemical signals and electrical signals. Therefore, artificial nerve conduits need to have electrical conductivity. Graphene (Gr) is an ideal three-dimensional electrical conductive material with conductivity that never disappears due to its high electron mobility at room temperature [8,9,10].

Perfect tissue engineering scaffolds should simulate natural tissue in the material composition, and also be identified with an extracellular matrix (ECM) in the structure such that cell adhesion and proliferation is promoted [11]. Nanofibrous scaffolds feature the above-mentioned characteristics, and also provide a high specific area, which helps to increase protein adsorption as a cell adsorption site [12]. Electrospinning technology is a reliable way to spin the nanofibrous membrane scaffolds needed for nerve conduits.

Sodium alginate (SA) can be electrospun into translucent bio-membranes that are smooth, tough, and water permeable. These membranes allow micromolecules to penetrate and intercept cells and bacteria [13]. However, SA membranes are easily decomposed and have low mechanical properties. Moreover, PVA membranes feature high toughness and ease of processing, and the combination of PVA and SA membranes improves the low mechanical properties. Hence, the modified PVA/SA composite membranes have great potential for medicinal purposes [14,15,16]. Adding nanofillers to nanofibers can improve the properties of nanofiber membranes [17]. One-dimensional carbon nanotubes (CNTs) are inserted into two-dimensional graphene sheets, which can connect the disconnected graphene sheet, as well as form steric hindrance among graphene sheets. The graphene/CNT composites have a large specific surface area and a three-dimensional grid structure, which facilitates the transmission of electrons. The synergistic effect of graphene and CNTs can efficiently build a conductive net in polymers, which subsequently improves the conductivity. Using a suitable method to prepare Gr/CNT composites, both materials demonstrate a synergistic effect that contributes to improve the properties of the composites [18,19,20]. Ionita et al. investigated the synergistic effect of carbon nanotubes and graphene on the high-performance cellulose acetate membranes as biomedical applications [21]. However, these studies employed a coating method to prepare membranes whose specific surface area was smaller. Therefore, this study uses an electrospinning method to fabricate artificial nerve conduits. Furthermore, the optimal ratio of carbon nanotubes and graphene has not been studied, and thus this study aims to study the optimal CNT/Gr ratio.

Continuous needleless dynamic linear electrospinning is used to prepare nerve conduits, which significantly improves the yields of industrial production over that of the conventional electrospinning [22]. Based on our previous study, we have optimized the processing parameters, including collection distance and voltage on PVA/SA/Gr nanofibrous membranes [23]. In this study, we focus on the study of the structural regulation of PVA/SA/Gr/CNT and effects of the content of graphene and carbon nanotubes on the conductivity of the nanofibrous membranes in order to provide a solid reference for nerve conduits.

## 2. Materials and Methods 

### 2.1. Materials

Polyvinyl alcohol (PVA, Mw = 84,000–89,000) was purchased from Changchun Chemical, Changchun, China. Sodium alginate (SA, purity: 90%), polyvinylpyrrolidone (PVP, K13-18, Mr: 10,000) and AR tritonX-100 were purchased from Shanghai Macklin Biochemical, Shanghai, China. Graphene (Gr, P-ML20) and multiwalled carbon nanotube (CNT, CF182C) were provided by Enerage, Taiwan. Deionized water was used in this study. 

### 2.2. Preparation of Nanofibrous Membranes

The optimal type and content of the dispersant were determined and used to formulate graphene and carbon nanotube solutions. Afterward, PVA was added and mixed. A multi-parameter tester was used to measure the conductivity, thereby determining the optimal Gr/CNT ratio to yield the highest conductivity.

**Figure 1:**
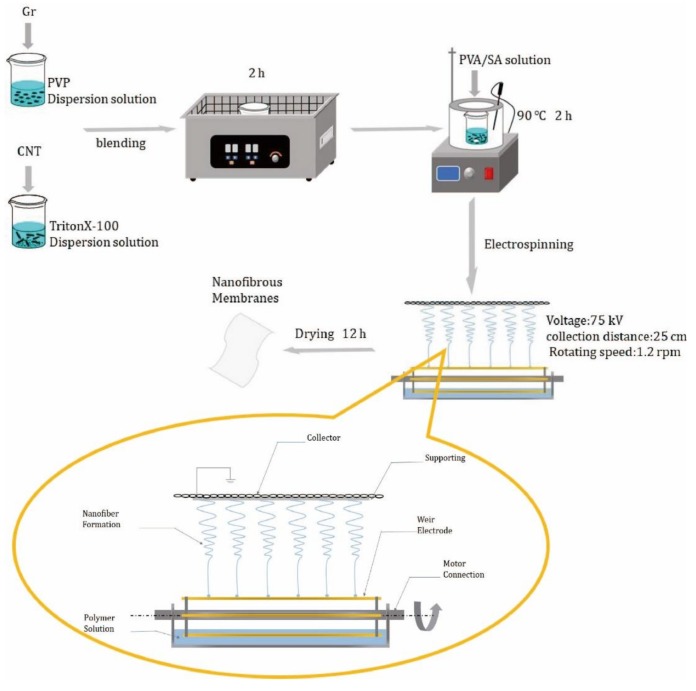
Process of the preparation of linear electrospinning nanofiber membranes.

First, graphene (0.05 wt%) and carbon nanotubes (0.02 wt%) were added to deionized water. Afterward, 1 wt% PVP was added to the Gr solution, while 0.5 wt% of tritonX-100 was added to the CNT solution. The solutions were stirred using a mechanical mixer for 2 hours. Next, Gr and CNT solutions were blended at volume ratios of 10:0, 2:8, 4:6, 6:4, 8:2, and 10:0 for 2 hours using a 200 W ultrasound machine (040ST, SHENCHAOJIE, Shenchen, China). Then, 7.5 wt% PVA solution was added to the mixtures, which was mixed at 90 °C for 2 h using a collecting thermostatic heating magnetic stirrer. Second, 2 wt% of SA powders were added to deionized water and mixed at 90 °C for 2 h. Third, different Gr/CNT mixtures were added to the SA solution at a volume ratio of 9:1 for 2 h. Finally, different electrospinning solutions were electrospun into nanofibrous membranes. The collection distance was 25 cm, the spinning voltage was 75 kV, and the rotating speed was 1.2 rpm [23]. The whole process of the preparation of linear electrospinning nanofiber membranes is shown in Figure 1.

### 2.3. Characterizations of Nanofibrous Membranes

A scanning electron microscope (TM3030, HITACHI, Tokyo, Japan) was used to measure the morphology of nanofibers. A bundle of 100 fibers in the SEM images were used to find the average diameter and diameter distribution. Fourier Transform Infrared (FTIR) spectrometer (NICOLET iS10, Thermo Fisher Scientific, Waltham, MA, USA) is used to analyze the functional group of nanofibrous membranes. The surface resistance instrument (RT-1000, OHM-STAT, Static Solutions Inc, Hudson, USA) is used to measure the surface resistivity, to study the influences of Gr and CNT on the conductivity and the conduction threshold of nanofibrous membranes. Raman spectrometer (XploRA PLUS, Horibagon, Tokyo, Japan) was used to analyze nanofibrous membranes at a laser wavelength of 473 nm in a range of 200–3200 cm^−1^. In order to investigate the surface characteristics, the water contact angle of the sample was measured using a surface contact angle instrument (JC2000DM, Shanghai Zhongchen Digital Technic Apparatus, Shanghai, China). The water contact angle on five samples in the first second was measured using Image-Pro Plus 6.0 (Media Cybernetics, MA, USA) in order to have an average. A differential scanning calorimeter (DSC200F3, NETZSCH, Bavaria, Germany) was used to analyze the pattern of melting points of membranes in a nitrogen atmosphere. Membranes were heated to 240 °C at increments of 10 °C/min. A thermogravimetric analyzer (TG 209F3, NETZSCH, Bavaria, Germany) was used with measurements done in a nitrogen atmosphere. Samples were heated to 700 °C at increments of 10 °C/min.

## 3. Results and Discussion

### 3.1. Conductivity of Gr and CNT Solutions

**Figure 2:**
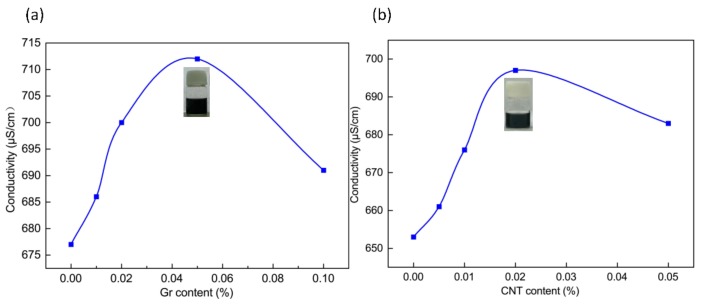
Conductivity of different concentrations of (**a**) Gr-PVA solution and (**b**) CNT-PVA solution.

Figure 2 shows the conductivity of Gr and CNT solutions with different concentrations. Regardless of whether it was a Gr or CNT solution, the conductivity increased and then decreased as the concentration increased. The highest conductivity occurred when the Gr solution had a concentration of 0.05 wt% or when the CNT solution had a concentration of 0.02 wt%. The results may be ascribed to the fact that an increase in the nanofillers caused agglomeration, which was detrimental to the effect of dispersion [24].

### 3.2. Morphology of Nanofibrous Membranes

**Figure 3:**
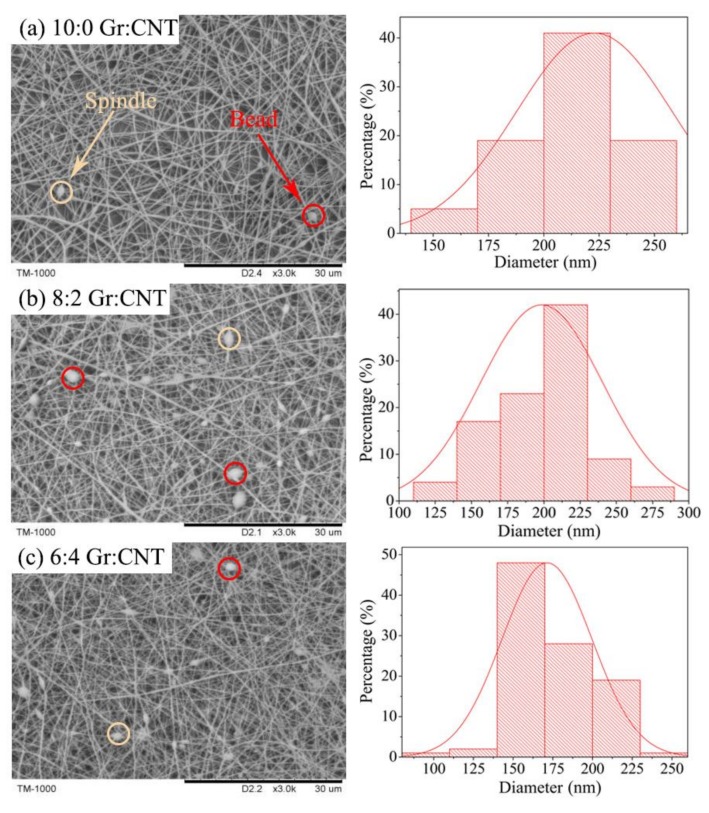

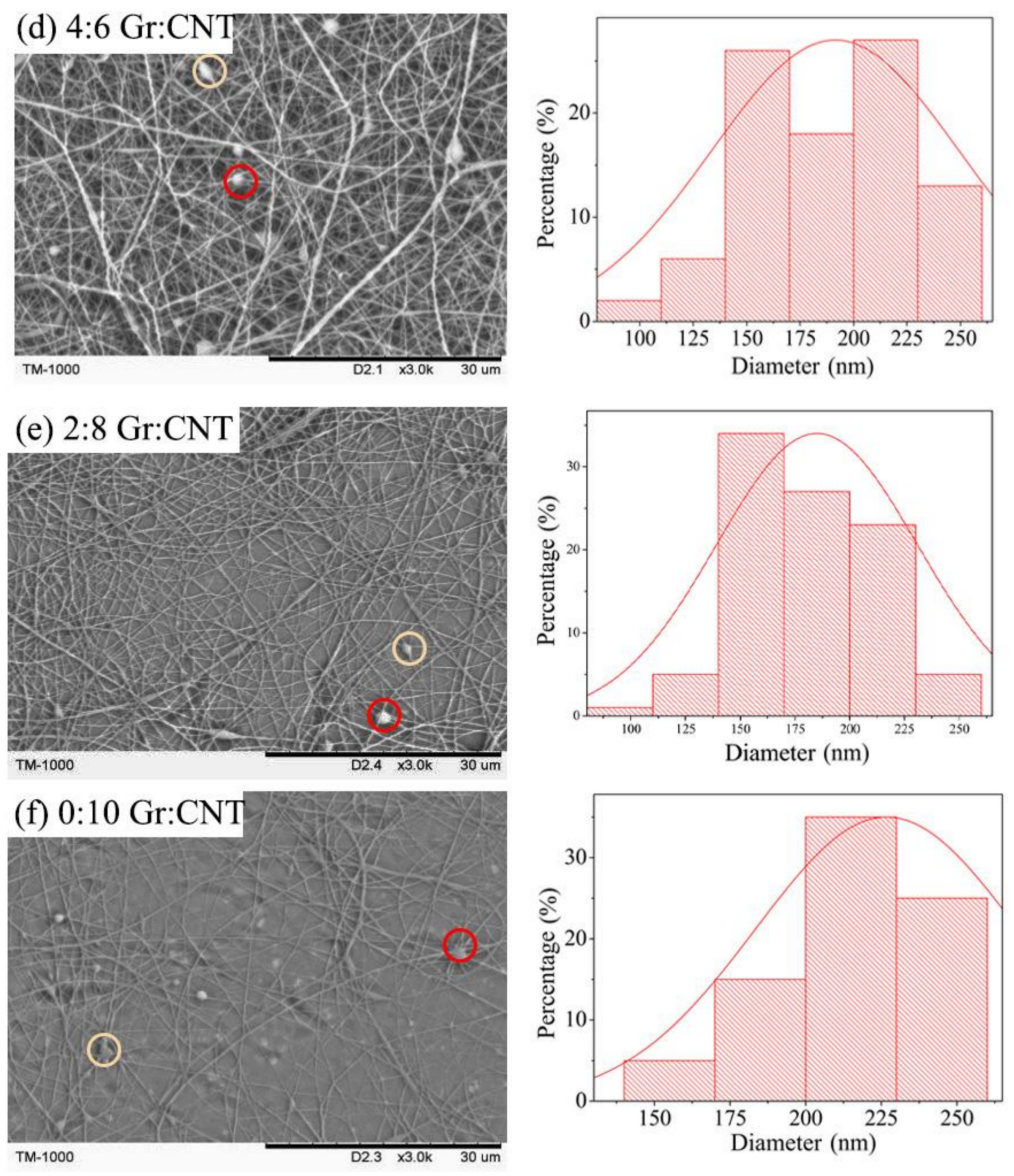
SEM images and diameter of PVA/SA/Gr/CNT nanofibers membranes with the Gr-CNT ratios of (**a**) 10:0, (**b**) 8:2, (**c**) 6:4, (**d**) 4:6, (**e**) 2:8, (**f**) 0:10.

SEM images in Figure 3 show that regardless of the Gr-CNT ratio, the resulting nanofibers were bead- and spindle-like. The beaded fibers occurred due to the presence of Gr and CNT. In the electrospinning process, Gr and CNT with a large particle size were expanded with PVA solution as a result of the electric field, and finally adhered to the PVA nanofibers [25]. By contrast, the spindle-like fibers were formed because Gr and CNT were not evenly distributed in the electrospinning solution, which subsequently caused the difference in the conductivity in local areas. A portion of droplets with higher conductivity was more greatly affected by the electric field force, and was thus not fully expanded to be collected on the collector [26]. Incorporation of Gr and CNT led to the agglomeration of a nanofiller, which might have caused the reduced mechanical properties and electrical conductivity of the nanofibrous membrane. The effect of nanoparticle distribution on the improvement of nanohybrid properties due to the strong interaction between the filler and matrix was similarly reported previously [27]. As a result, the needleless dynamic linear electrospinning was able to form finer nanofibers with an average diameter of 200 nm. When Gr and CNT blended with a 6:4 *v*/*v* ratio, the average diameter was about 171 nm. Moreover, the spinnability decreased as the CNT content increased, which was due to the viscosity and surface tension of the spinning solution as displayed in Table 1. It was found that as the CNT ratio was gradually increased, the surface tension of the spinning solution gradually decreased. Low surface tension of the spinning solution generated a finer jet flow, and it was difficult for the lower viscosity electrospinning solution to form a stable jet flow, both of which caused the fiber morphology deterioration [28]. 

### 3.3. FTIR Spectrum Analyses

**Figure 4:**
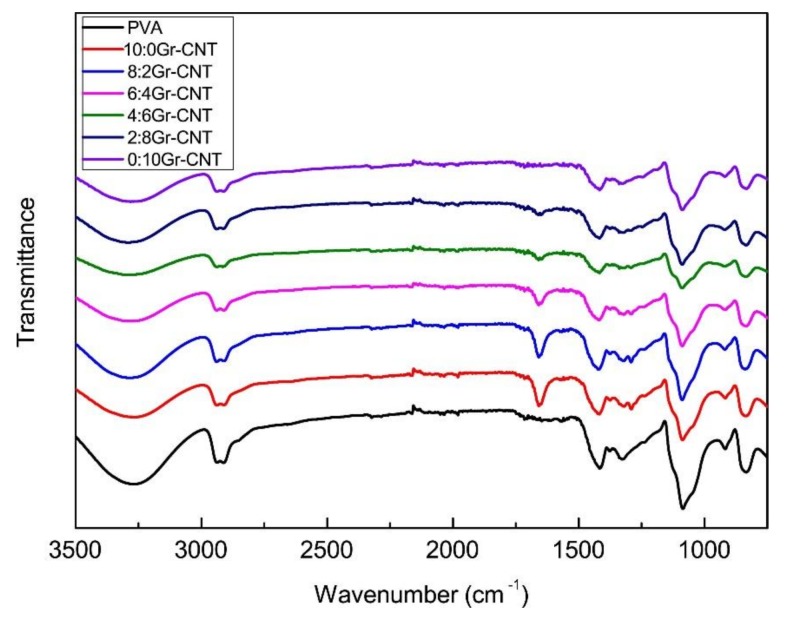
FTIR spectrum of PVA/SA/Gr/CNT nanofibrous membranes as related to Gr-CNT ratios.

Figure 4 shows that different Gr-CNT ratios form membranes that have a similar spectrum. The characteristic peaks at 2850 cm^−1^ correspond to the absorption peak caused by the stretching vibration of O–H and C–H from PVA, SA, and the residual water. The absorption peak triggered by C=O of –COOH occurred at 2210.9 cm^−1^, the characteristic peak caused by the covalent bond between carbon atoms and hydroxyl groups occurred at 2100cm^–1^, the absorption peak caused by stretching vibration of C=C occurred at 1630 cm^−1^, the absorption peak caused by the stretching vibration of C–O from C–O–C occurs at 1119.8 cm^−1^ [29]. Noticeably, the characteristic peaks at 1058 cm^−1^ were strengthened due to a greater content of Gr, which indicates that Gr was successfully blended in nanofibers and even mixed with CNT. The characteristic peaks of all curves occurred in the same position, which suggests the absence of new functional groups. Therefore, the presence of graphene and CNT and the Gr-CNT ratio did not cause a chemical reaction of mass in the solution.

### 3.4. Surface Resistivity

**Figure 5:**
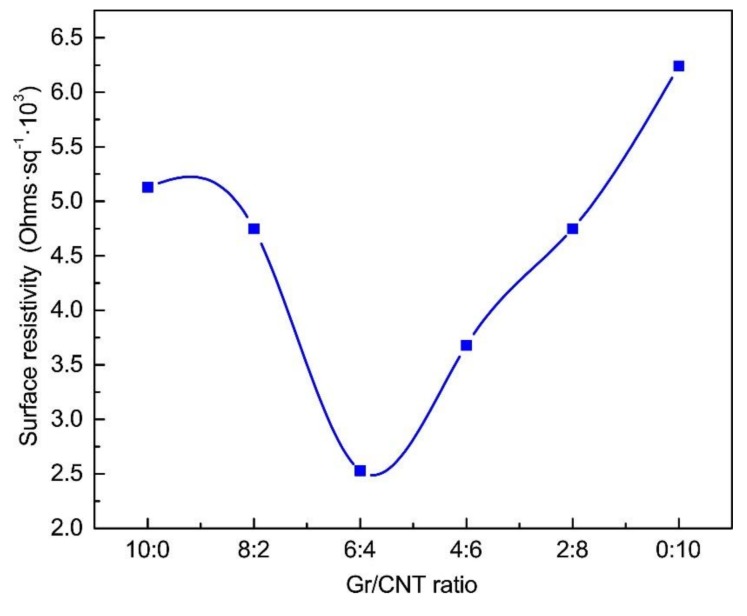
Surface electric resistivity of PVA/SA/Gr/CNT nanofibrous membranes as related to the Gr-CNT ratios. (Control: The surface resistivity of the PVA/SA nanofiber membrane was 7.58 × 10^9^ Ω/m^2^).

Figure 5 shows that, compared to pure PVA nanofibrous membranes, the PVA/SA/Gr/CNT nanofibrous membranes had a significantly lower surface resistance, which decreased from 10^9^ to 10^3^ Ω/m^2^. It indicates that the presence of Gr and CNT can improve the conductivity of nanofibrous membranes effectively. Gr and CNT can bridge conductive paths inside the nanofibrous membranes, and the conductivity is thus improved [30,31]. The surface resistance of PVA/SA/Gr/CNT nanofibrous membranes first increased and then decreased with an increase in the content of the CNT solution. Moreover, PVA/SA/Gr/CNT nanofibrous membranes had a lower surface resistance than nanofibrous membranes with a single nanofiller, which similarly meant a greater conductivity. In particular, with a Gr-CNT ratio of 6:4, PVA/SA/Gr/CNT nanofibrous membranes had the lowest surface resistivity of 2.53 × 10^3^ Ω/m^2^ and the highest conductivity. The result proves that graphene and CNT have a synergistic effect, which is advantageous for the conductivity of nanofibrous membranes. A study shows that reducing the medium conductivity results in lower viability of the cells in this medium [32].

### 3.5. Raman Spectrometry

**Figure 6:**
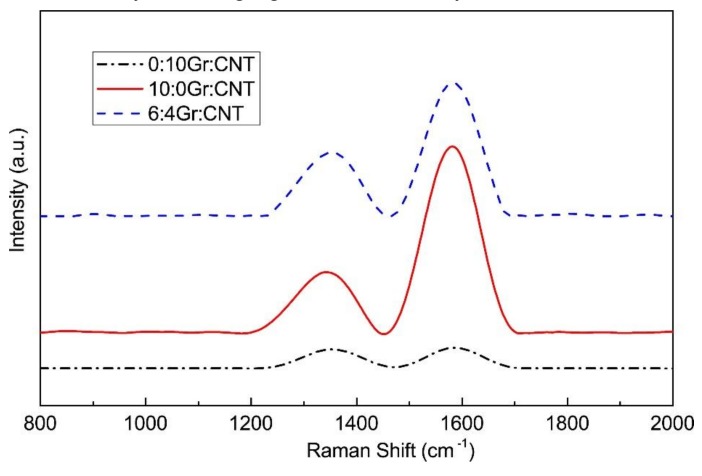
Raman spectrometry of PVA/SA/Gr/CNT nanofibrous membranes as related to Gr-CNT ratios.

In order to verify the synergistic effect of CNT and Gr, the structure of PVA/SA/Gr/CNT nanofiber membrane was analyzed using Raman spectroscopy. Raman spectrometry characterizes structures and components of carbon materials, which helps to acquire the bonding state of carbon materials [33]. The strength of characteristic peaks in the D and G bands can be measured to investigate the order level of Gr and CNT. The D band is the characteristic peak that is the defect of the carbon atom lattice, which reflects the disorder and defects of carbon materials. The characteristic G band peak represents the in-plane stretching vibration of carbon atom sp^2^ hybridization, reflecting the level of symmetry and graphitization of the carbon nanotube wall atoms and graphene [34,35]. Regardless of whether it was Gr/SA nanofibrous membrane, CNT/SA nanofibrous membrane, or PVA/SA/Gr/CNT nanofibrous membrane at a Gr-CNT ratio of 6:4, there were significant characteristic peaks at 1350 cm^−1^ (i.e., D band) and 1580 cm^−1^ (i.e., G band) [36,37]. The ratio of D and G bands (ID/IG) is used to measure the level of defects. A greater ID/IG means that the structure has more defects. Figure 6 shows that the ratio of D and G bands was A = I_D1_/I_G1_ = 0.33 for Gr/SA nanofibrous membranes, B = I_D2_/I_G2_ = 0.92 for Gr/SA nanofibrous membranes, and C= I_D3_/I_G3_ = 0.48 for PVA/SA/Gr/CNT nanofibrous membrane with a Gr/CNT of 6:4, and can be ranked as A < C < B. The results show that the addition of Gr and CNT provides carbon materials with more defective structures. Hence, the electrons can be more easily transmitted in PVA/SA/Gr/CNT nanofibrous membranes, thereby obtaining a greater conductivity [38].

### 3.6. Water Contact Angle

**Figure 7:**
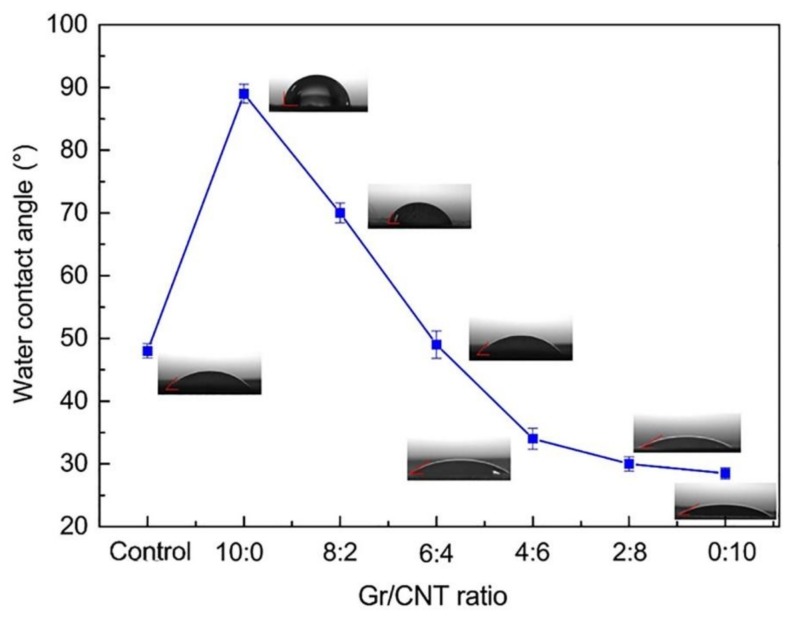
Water contact angle of PVA/SA/Gr/CNT nanofibrous membranes as related to Gr-CNT ratios. (Control: PVA/SA nanofibrous membranes).

Figure 7 and Table 2 respectively shows the differences and values in water contact angle of PVA/SA/Gr/CNT nanofibrous membranes as related to Gr/CNT. More Gr caused a larger contact angle for nanofibrous membranes. The water contact angle of PVA/SA nanofibrous membranes was smaller, which was 48°. PVA has considerable hydrophilic –OH groups and SA has –COOH groups, which results in a high affinity with water molecules and provides nanofibers with greater moisture absorption capacity [39]. Furthermore, the presence of Gr increases the water contact angle, and there are thus significant differences in the nanofibrous membranes containing different contents of Gr. When the Gr-CNT ratio was 10:0, the water contact angle increased to 89° and the PVA/SA/Gr nanofibrous membranes showed good hydrophobicity. When the CNT ratio gradually increased, the contact angle was gradually decreased. This may be due to the fact that a decrease of the content of Gr, which has a strong hydrophobicity, resulted in an increase of hydrophilicity of the nanofibrous membrane. Moreover, the addition of graphene and carbon nanotubes also gave the nanofibers with a rough surface. According to the Wenzel equation (Equation (1)), the relationship between the wetting angle of a rough interface (i.e., θ*) and a wetting angle (i.e., *θ*) can be presented as in Equation (1). When *γ* > 1 and the angle is smaller than 90°, the nanofibers have a rougher surface and a smaller surface contact angle [40,41].

(1) $$\cos\theta^{*}=\frac{f\left(\gamma_{CL}+\gamma_{CS}\right)}{\gamma_{SL}}=f\cos\theta$$

### 3.7. Thermal Property Analyses

**Figure 8:**
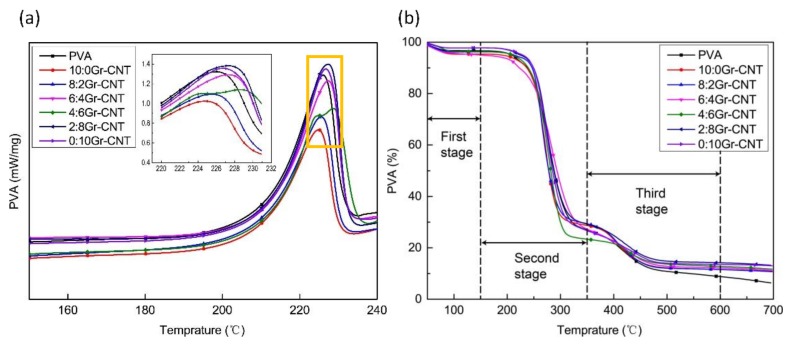
Characterization of thermal properties of PVA/SA/Gr/CNT nanofiber membranes: (**a**) DSC of nanofibrous membranes as related to Gr-CNT ratios, and (**b**) TG of nanofibrous membranes as related to Gr-CN ratios.

Figure 8a shows that the PVA/SA/Gr/CNT nanofibrous membranes exhibited similar DSC curves with a molten peak within 220–230 °C. The molten peak of PVA nanofibrous membranes occurred at 226.1 °C. Increasing the content of Gr caused the molten peak to shift toward high temperatures. Conversely, when the Gr-CNT ratio was 4:6, the molten peak shifted toward low temperatures. With Gr being dispersed, –COOH and –OH were formed over Gr’s surface. When the content of Gr increased, the –OH of PVA molecular chains and the oxygen functional groups of Gr were combined via hydrogen bonds. The drastic interaction adversely affected the activity of PVA and SA molecular chains [42]. Nevertheless, the excessive content of Gr causes agglomeration and the molten peak was comparatively decreased.

Figure 8b shows the TG curves of PVA/SA/Gr/CNT nanofiber membranes as related to Gr/CNT. The corresponding results are displayed in Table 3. The TG curves directly show three weight loss stages. The first stage occurred at 50–100 °C, which was primarily due to the evaporation of the residual water or solvents in the nanofibrous membranes, and the weight loss was small. The second stage shows the degradation peak at 150–350 °C, which was the main stage of thermal degradation caused by the breakage of PVA and SA macromolecular chains, and the weight loss was 55–75% [43,44]. Based on the slope, the maximum decomposition rate also occurred at the same stage. The third stage of weight loss occurred at 350–600 °C, which reflected the chain-breaking and cyclization reactions of PVA and SA molecules. The weight loss rate was 10–20%. The residual mass was 10–14% for PVA/SA/Gr/CNT nanofibrous membranes and 6.3% for PVA/SA nanofibrous membranes, which suggests that Gr and CNT were not easily decomposed at 700 °C.

## 4. Conclusions

The conductivity of PVA/SA/Gr/CNT nanofibrous membranes first increased and then decreased. The highest conductivity occurred when the graphene solution had a concentration of 0.05 wt% or when the CNT solution had a concentration of 0.02 wt%. Using the needleless dynamic linear electrospinning successfully produced PVA/SA/Gr/CNT nanofibrous membranes with an average fiber diameter of 200 nm and a production yield of 1.25 g/h, which is greater than using the traditional electrospinning process. The Gr-CNT ratios affected the properties of nanofibrous membranes, and a decrease in graphene reduced the amount of bead- and spindle-shaped nanofibers. Moreover, the presence of graphene and carbon nanotube improved the thermal stability, hydrophobicity, and conductivity. Specifically, a Gr-CNT ratio of 6:4 provided the nanofibrous membranes with an optimal synergistic effect and the highest conductivity. Therefore, the proposed PVA/SA/Gr/CNT nanofibrous membranes have a promising future in the tissue engineering field.

## Figures and Tables

**Table 1:** Viscosity and surface tension of spinning solution.

**Gr: CNT**	0:0	10:0	8:2	6:4	4:6	2:8	0:10
**Viscosity (mPa^.^ s)**	214	221	219	198	190	207	193
**Surface tension (mN /m)**	42.7	41.2	37.6	35.2	31.5	30.9	30.1

**Table 2:** Contact angle and standard deviation of nanofiber membranes.

**Gr: CNT**	0:0	10:0	8:2	6:4	4:6	2:8	0:10
**Contact angle (°)**	48 ± 1.14	89 ± 1.52	70 ± 1.58	49 ± 2.17	34 ± 1.67	30 ± 1.14	28.5 ± 0.89

**Table 3:** DSC and TG results of nanofiber membranes.

Gr: CNT	T_5%_ (°C)	T_MAX_ (°C)	Loss in the First Stage (%)	Loss in the Second Stage (%)	Residual Mass (%)
0:0	208.6	276.6	96.66	26.92	6.34
10:0	190.8	271.5	95.67	28.68	10.84
8:2	212.9	265.0	94.98	29.35	10.70
6:4	216.4	264.1	96.36	27.25	11.09
4:6	210.2	281.7	96.36	23.34	11.67
2:8	224.7	275.1	97.78	29.18	13.22
0:10	229.3	275.5	97.79	26.65	12.99
